# Constructed wetlands combined with microbial fuel cells (CW-MFCs) as a sustainable technology for leachate treatment and power generation

**DOI:** 10.1039/d4ra04658g

**Published:** 2024-10-11

**Authors:** Isni Arliyani, Md Tabish Noori, Muhammad Imam Ammarullah, Bieby Voijant Tangahu, Sarwoko Mangkoedihardjo, Booki Min

**Affiliations:** a Department of Environmental Engineering, Institut Teknologi Sepuluh Nopember Surabaya 60111 East Java Indonesia; b Bioinformatics Research Center, INBIO Indonesia Malang 65162 East Java Indonesia; c Department of Environmental Science and Engineering, Kyung Hee University Yongin 17104 Gyeonggi Republic of Korea bkmin@khu.ac.kr; d Department of Mechanical Engineering, Faculty of Engineering, Universitas Diponegoro Semarang 50275 Central Java Indonesia; e Undip Biomechanics Engineering & Research Centre (UBM-ERC), Universitas Diponegoro Semarang 50275 Central Java Indonesia; f Bioengineering and Environmental Sustainability Research Centre, University of Liberia Monrovia 1000 Montserrado Liberia imamammarullah@gmail.com

## Abstract

The physical and chemical treatment processes of leachate are not only costly but can also possibly produce harmful by products. Constructed wetlands (CW) has been considered a promising alternative technology for leachate treatment due to less demand for energy, economic, ecological benefits, and simplicity of operations. Various trends and approaches for the application of CW for leachate treatment have been discussed in this review along with offering an informatics peek of the recent innovative developments in CW technology and its perspectives. In addition, coupling CW with microbial fuel cells (MFCs) has proven to produce renewable energy (electricity) while treating contaminants in leachate wastewaters (CW-MFC). The combination of CW-MFC is a promising bio electrochemical that plays symbiotic among plant microorganisms in the rhizosphere of an aquatic plant that convert sun electricity is transformed into bioelectricity with the aid of using the formation of radical secretions, as endogenous substrates, and microbial activity. Several researchers study and try to find out the application of CW-MFC for leachate treatment, along with this system and performance. Several key elements for the advancement of CW-MFC technology such as bioelectricity, reactor configurations, plant species, and electrode materials, has been comprehensively discussed and future research directions were suggested for further improving the performance. Overall, CW-MFC may offer an eco-friendly approach to protecting the aquatic environment and come with built-in advantages for visual appeal and animal habitats using natural materials such as gravel, soil, electroactive bacteria, and plants under controlled condition.

## Abstract 2

The physical and chemical treatment processes of leachate are not only costly but can also produce harmful by-products.

## Graphical abstract

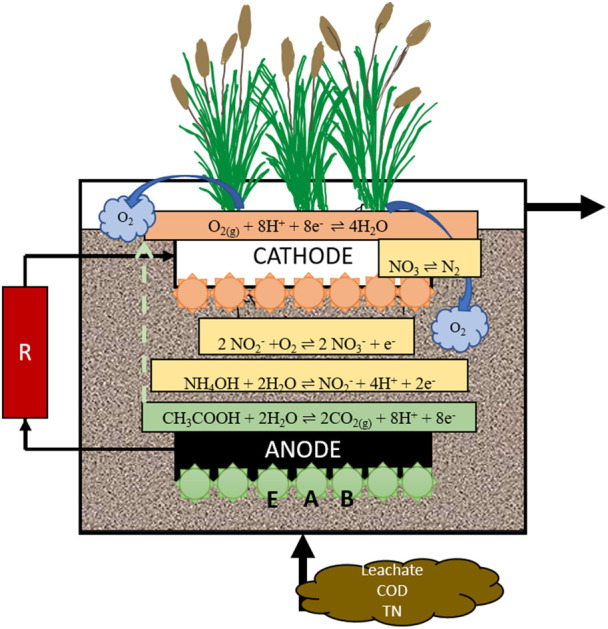

## 1 Introduction

To dispose solid waste, most developing nations adopt an early stage of landfilling in the form of open dumping.^1^ Concerns in landfill design and operation include landfill leachate (LL), emission gas, slope stability, and odor management.^2^ Open dumps are places where there is no environmental protection or oversight. Designed landfills are distinguished by correct site selection and design, trash compaction, applying daily cover and leachate, and gaseous and odour systems. Designed landfills include onsite leachate treatment and a post-closure management plan.^3^ Pollutants in leachate are classified into four types: dissolved organic matter (organic carbon and fatty acids), inorganic materials (chlorides, ammonium, phosphorous, and nitrates), toxic metals (copper, zinc, lead, and mercury), and xenobiotic organic compounds (XOCs) (benzene, phenols, and phthalates).^4,5^ Raw LL has a high concentration of pollutants.^6,7^ If not collected and treated, it has a great potential to pollute surrounding soil and groundwater.^8,9^

Leachate from various sources, such as municipal waste, industrial waste, industrial landfills, and solid inert residue landfills, presents unique challenges due to their differing compositions and pollutant concentrations. Recent advancements in landfill leachate treatment technologies have provided several methods, each with distinct advantages and disadvantages. Traditional methods, while effective, often fall short in terms of environmental sustainability and economic feasibility. Among the various processes to treat LL, constructed wetlands (CWs) have proven effective and economical. Constructed wetland (CW) systems utilize vegetation that supports water purification processes for treatment.^10^ This technology is low-cost and uses a combination of plants, microorganisms, and soil/media to treat pollutants.^11,12^ CWs provide symbiotic physical processes, including filtration; physicochemical adsorption; ion exchange; chemical decomposition; precipitation; and microbial reactions such as biodegradation, ammonification, nitrification, and denitrification.^13^ Previous studies have shown that various inorganic and organic contaminants are removed through these mechanisms in CWs.^14^ CWs are advantageous over other methods based on environmental attributes, such as their ability to integrate into natural landscapes and provide additional ecosystem services.

However, the direct treatment of leachate using CW is applicable but challenging.^15^ Single-process treatments often struggle due to high pollutant concentrations that inhibit the process.^16^ CWs can produce a favorable chemical composition to enhance pollutant degradation.^17–19^ The performance of CWs depends on factors such as design, plants, media, hydraulic retention time, weather, and more.^20–22^ Combining microbial fuel cells (MFC) with CWs (CW-MFC) is a relatively new approach for treating pollutants and generating electricity.^23,24^

Integrated CW technologies, such as CW-MFC systems, offer a sustainable solution for leachate treatment by leveraging the synergistic effects of CWs and MFCs. CW-MFC systems combine the biological processes of CWs with the electrochemical processes of MFCs, enhancing pollutant degradation and electricity generation. This integration allows for the treatment of complex leachates while producing renewable energy. The design parameters, such as the choice of plants, electrode materials, and hydraulic configurations, significantly influence the performance of CW-MFC systems.

Electrochemical technology, which uses reduction and oxidation processes, has also shown promising results for leachate treatment.^25^ W-MFC treatment has been researched as a promising technology for treating diverse wastewater and producing electricity. Pollutants in multiple wastewaters can be reduced through the combination of plants, bacteria, fillers, and electrochemical redox processes in CW-MFC systems. Microorganisms in CW-MFC systems degrade organic pollutants to generate electrons and protons, occasionally converting the chemical energy of organic contaminants into electrical energy.^26^ A CW-MFC includes electrodes (anode and cathode) separated by gravel, sand, and soil (media CW), proton exchange membrane (PEM), and fibrous materials.^27^ CW-MFCs operate under both anaerobic and aerobic conditions, facilitating redox transformations.^28,29^ Aerobic conditions form near the CW-MFC surface (cathode location), while anaerobic conditions occur at the reactor bottom (anode location).^30–32^ Previous research on treating municipal sewage using CW-MFCs showed significant reductions in nitrogen, organic matter, and phosphorus.^33,34^ Studies using synthetic wastewater reported organic and nitrogen removal percentages of 73–98% and 50–85%, respectively, similar to leachate contents.^35,36^ CW-MFCs can eliminate biological oxygen demand, chemical oxygen demand, ammonium nitrogen, total nitrogen, and phosphorus, with removal percentages ranging from 35 to 76%, 22 to 76%, 10 to 90%, 0.2 to 71%, and 38 to 92%, respectively. Voltage production varied between 4 and 152 mV, with the highest power density between 50 and 527 mW m^−3^.^37^

Recent years have seen increased attention to leachate treatment, a complex and environmentally significant wastewater stream. Various treatment methods have been explored, focusing on innovative and sustainable approaches. This review differentiates and highlights the unique perspective provided by the literature on leachate treatment in CW-MFC systems. While numerous studies have examined leachate treatment methods and CW-MFC for wastewater treatment, the distinctiveness of the CW-MFC approach lies in its integration of biological processes within a CW framework, coupled with the harnessing of MFC technology. This research sets the stage for a comprehensive exploration of how CW-MFC compares to other studies, offering a nuanced understanding of its design, electricity production, advantages, and future potential as a promising solution for leachate treatment.

New pollution characteristics of leachate due to technological advancements and household applications can include hazardous compounds, insecticides, heavy metals, and persistent organic pollutants.^38,39^ Review papers have discussed leachate treatment and electrode materials,^40,41^ bioenergy generation mechanisms,^42,43^ and comparisons between CW and CW-MFC.^44^ To fill the gap, this review focuses on leachate pollutants, design, electrode materials, removal efficiencies, plant types, CW-MFC mechanisms, future directions, and challenges. Compared to other reviews, this research will provide a comprehensive report on these aspects.

## 2 Source of landfill leachate and characteristics

Leachate is the term for the liquid that comes from the landfill and contacts the waste pile to dissolve the dissolved material.^45^ Depending on the type of waste in different regions of the landfill, it will produce different leachate qualities. LL is characterized as either young (<5 years), mature (5–10 years), and old (>10 years).^46–48^ A high BOD/COD ratio (greater than 0.5) in leachate produced in young landfills is an indicator of leachate biodegradability. Amino acids are found in young LLs because they are released when organic molecules break down. Leachate from old landfills is rich in ammoniacal nitrogen due to hydrolysis and fermentation of the nitrogen fraction of biodegradable substrates. Changes in organic matter and ammoniacal nitrogen over time can significantly impact leachate treatment. Regardless of the age of the landfill, leachate consistently contains a variety of life-threatening and ecologically damaging toxins.^49^ Waste sources can be divided into three categories: solid inert residue (SIR), industrial solid waste (ISW), and municipal solid waste (MSW). Over the past year, the average MSW generation rate per capita in Qatar has reached 1.5 kg per person per day.^50^

### 2.1 Municipal waste leachate

Municipal solid waste (MSW) is defined as debris, solid refuse, and rubbish that is produced within the boundaries of a district and a municipality, regardless of where it is generated.^45^ The population has the greatest impact on the volume of MSW.^51^ Since each resident in each area will be unique, non-resident areas like institutional or business districts and resident regions can be distinguished from one another.^52^ The research on MSW landfill leachate has shown that it is a major source of worry for new and developing compounds. Perfluorosurfactants (PF), such as perfluoro octane sulfonate (PFOS, C_8_F_17_SO_3_H) and perfluorooctanoic acid, are another new class of developing surfactants (PFOA, C_7_F_15_COOH).^53^ They have attracted interest because of their distinct chemistry (possible water and oil repellent and surface tension lowering), persistence, bioaccumulation, and hazardous consequences. These are also the most common anionic PF surfactants and organophosphates found in the environment.^54^ An early investigation found sulphonamide antibiotics in groundwater near an MSW in Denmark. Sulphonamide concentrations in LL reached 18 mg L^−1^, which is substantially higher than is typically found in raw urban and agricultural wastewater sources.^55^

Due to geographic location, climate conditions, food habits, and cultural and religious events, the content of the MSW may change seasonally.^56^ The physical classification of waste organic matter and aggregates includes paper and plastic. The processing capability of biological treatment facilities is decreased when aggregate components are mixed with recyclable resources. The granulometry of aggregate material, which ranges from the smallest to the largest, makes separation from the waste challenging and increases processing costs for material recycling plants. For the capping and filling process in landfills, sewage sludge, dust, and sand must be present and assembled separately. The MSW's moisture content ranged from 28% to 35%. Data on moisture content provide insight into pre-treatment technologies being developed for efficient waste treatment. Between 39% and 43% of solids were observed to be volatile.^56^ Reduce, reuse, recycle, recover, and then landfill disposal are the principles used to handle MSW.^57,58^ Unsafe waste management practices can lead to environmental issues, one of which is water pollution from leachate.^1,59^

Due to the significant amounts of organic matter (such as carboxylic acids and dissolved solids), toxic chemicals, inorganic salts, heavy metals, ammonia, minerals, and xenobiotic organic compounds in MSW leachate from city solid waste treatment facilities, landfills, anaerobic stoves, or compost piles poses serious environmental problems that require attention.^60,61^ The organic part of landfill filtrate is dominated by flame retardant or non-biodegradable chemicals, such as humic substances like humic acids.^62^ High amounts of these environmental contaminants are found in the filtrate derived from personal care products and home chemicals. Because it may leak into groundwater and produce biomagnification, LL is dangerous and characterized by both acute and chronic toxicity.^63^ Leachate from landfills frequently seeps into the soil, which is an issue for landfills worldwide. There are landfills and semi-controlled landfills, and if they are open, they might leak into low-lying coastal regions and contaminate the leachate flowing water. MSW seeps into groundwater or mixes with surface waters as a result of percolation, which can also be brought on by heavy rain and melting permafrost in polar regions.^58^ The concentration of leachate MSW is shown in Table 1.^64,65^

**Table 1:** MSW leachate concentration

Parameter	Concentration (mg L^−1^)
pH	6.08–8.38
Color	61
SS	22.3
Chloride	455
Ammonium	28.6–2380
TN	57.1
TP	6.3
COD	1075–23 680
Oil	0.44
Cr_VI_^+^	0.025
Hg	0.00004
Cd	0.001
BOD	350–11 300

### 2.2 Industrial landfill leachate (ISW)

Another category is ISW type, which includes all facilities that generate such solid waste, regardless of size and location, and are generated by an industrial-type facility.^45^ Pollution brought on by companies is a serious worry across the world, and of all industrial sectors, the food business has the most influence because of excessive waste output rates per unit of production.^66^ Recent reviews^67–76^ and scientific studies^77–79^ have reported on phytochemical/bioactive substances produced by the processing of product wastes. The groups of organic compounds that are most prevalent in this waste are carbohydrates (pectin and oligosaccharides, starch, cellulose, dietary fibres, monosaccharides, *etc.*), bisphenols (lignin, phenolic acids, flavonoids, tannins, ellagitannins, *etc.*), proteins, lipids, essential oils (*e.g.*, terpenoids, hydrocarbons, alcohols and aliphatics). An average of 10% more linked disposal of industrial solid waste, such as textile, dairy, and others, has greatly increased.^80^ The amount of these compounds in this ISW varies from a few mg to a few grams per kilogram of waste, and their commercial value varies from several euros to several thousand euros per kg of finished product, depending on the compound and the final purity after recovery.^81^

There is a significant number of diverse types of garbage in landfills, where complicated physical, chemical, and metabolic processes controlled by environmental circumstances result in plastic fragmentation into microplastics.^82^ Landfill leachate can also be a source of dangerous chemical compounds such as bisphenol A (BPA). Plasticizers are a very important group of LL aromatic pollutants, as plastics make up a large part of waste emissions. BPA is a plastic component also found in thermal paper and most epoxies.^83^ According to prior research, BPA might be released from various plastic items *via* diffusion, hydrolysis, and decomposition, depending on their physicochemical qualities^84^. van Praagh *et al.*^85^ collected data on the amounts of microplastics in leachate from 11 landfills in Finland, Norway, and Ireland. The primary aims of this study were to examine the effect of different treatment procedures on microplastic removal efficiency and to identify probable sources of emission. Previous studies of LL pollutants have emphasized toxic organic chemicals such as BTEX (benzene, toluene, ethylbenzene and xylene, chlorinated and aromatic hydrocarbons, *etc.*)^86^. He *et al.*^87^ investigated the presence of microplastics in 12 leachate samples collected from two closed and four active landfill sites in China. According to ref. 88, BPA leaks from goods that have been disposed of in landfills.^89^ investigated 10 landfill sites in Japan and discovered BPA values ranging from 1.3 to 17 200 μg L^−1^. In one study, BPA was commonly detected in LL at concentrations up to 6–17 mg L^−1^, with a mean concentration of 45.4 μg L^−1^.^90,91^

Previous research^92^ has shown that developing microcontaminants, such as nonylphenol, phthalate acids, and BPA, can leak from plastic materials during weathering or aging of microplastic particles. These chemicals are classified as endocrine disruptors, and as such, they have a harmful influence on water quality. These chemicals are classified as endocrine disruptors, and as such, they have a harmful influence on water quality. They can interact with other contaminants in aqueous medium due to their hydrophobic nature and large surface area.^93^ BPA is a plasticizer that, due to its unique physicochemical qualities, is employed as a raw ingredient in the creation of various industrial and consumer products. BPA is one of the compounds that is widely used around the world.^94^

Some countries produce hundreds of millions of tons of different industrial trash each year as developing nations with strong economic growth^95^ and dispose of it in landfills. The leachate concentration of ISW in Northern Portugal, which collects a lot of trash from industrial processes, including sludge, ash, fibre cement, textile waste, plastic waste, metal, biodegradable waste, paper, cardboard, and wood from municipal wastewater treatment plants, is shown in Table 2.^90,96,97^

**Table 2:** ISW leachate concentration

Parameter	Concentration (mg L^−1^)
pH	8.2
Color	3800
SS	219
Chloride	1247
Ammonium	834
TN	1160
TP	4.3
COD	2667
Oil	—
Cr_VI_^+^	0.2
Hg	—
Cd	<0.05
BOD	245
Bisphenol A (BPA)	17

### 2.3 Solid inert residue landfill leachate

The distribution of SIR in the regions varies noticeably, and these variations are directly tied to the regional industrial structures. Geographically, Eastern China is the main generator of SIR from industry. A SIR released 32 292 million tons of garbage in 2017, or 46.7% of the overall production in China. The central/southwest portion of the area came in second with 16.2% and 13.1%, respectively. Regarding treatment capacity, cities' ability to treat SIR and disassemble big and medium amounts of e-waste greatly outpaced the quantity of treatment.^80^ Before being dumped in a landfill, SIR, such as bottom ash, is frequently separated from ferrous particles and occasionally from aluminium. In SIR landfills in other nations, bottom and fly ashes are still disposed of together. Almost all organic materials oxidize during combustion. Fly ash has a significantly larger leachable quantity of heavy metals than bottom ash. Several inorganic components lose their chemical connections during combustion due to the high temperatures.^98^

General SIR is actually a potential complex mixed resource comprising non-metal oxides, metal oxides, carbonates, and other materials.^99–101^ SiO_2_, Al_2_O_3_, Fe_2_O_3_, CaO, MgO, NaO, KO, and organic matter make up the majority of the composition's chemicals. Chemical composition, crystal structure, breakdown characteristics, particle size distribution, and mineral manufacturing methods all have a significant role in the physical and chemical features of waste. Industrial solid waste with a SiO_2_ component, such as sediments, carbon deposits, fly ash, and red soil, is often referred to as SIR.^102,103^ In order to prepare various kinds of porous materials to effectively and affordably transform SIR into porous materials, it is required to specify the physical and chemical substance qualities and degree of toxicity of various forms of SIR.^104^

About 71% of SIR is dumped in landfills around the world.^105^ Hazardous substances are frequently found in SIR, including certain batteries, paints, mercury-containing garbage, medications, auto care products, and a variety of other items.^106,107^ The SIR is dumped in landfills that also contain lead and mercury. To prevent these freshwater contaminants from entering groundwater aquifers, a large portion of these poisons must be adequately handled in landfills.^108^ Groundwater is seriously threatened by the leachate generation and poor management practices associated with unmanaged landfills, particularly open dumps. A polluted liquid called SIR leachate is released from SIR waste.^109^ Since leachate may infiltrate and contaminate groundwater, it needs to be carefully controlled. From SIR, the samples included 101 of the 190 chemicals analysed, with the levels of chemicals in each final leachate sample ranging from 2 nanograms per litter (ng L^−1^) to 17 200 000 mg L^−1^. Toxic items in landfills can contain these chemicals.^109^

Leachate is collected and treated onsite in contemporary landfills using physical/chemical treatment and biological treatment.^53^ Nevertheless, because LL treatment was designed primarily to focus on conventional water quality such as COD, BOD, ammonium, and so on,^110^ treated leachate still included significant amounts of pharmaceuticals and personal care products (PPCP). In Taiwan, for example, the average removal effectiveness of PPCPs in LLs was 15% for diclofenac (DF), gemfibrozil (GF), and carbamazepine (CBZ) over various treatment methods.^111,112^ About 5000 μg kg^−1^ of thiabendazole was found in LL together with CBZ. Similarly, CBZ in LL can contaminate groundwater through surface drainage from the point of use.^113^ The second major source of CBZ contamination is the groundwater–surface interface. This interface acts as an indirect way of mixing surface and groundwater through runoff and down-migration mechanisms.^113^ A high concentration of CBZ found in LL can easily contaminate groundwater.^114,115^

Ketoprofen and gemfibrozil are two carboxylic PPCPs that may be removed by activated sludge, although amide PPCPs (such as CBZ and crotamiton) are less effective.^116,117^ Industrial sludge, sewage treatment plant (ETP) sludge, contaminated drums, contaminated bags, and other hazardous waste produced by more than 2000 industries, including the pesticides and insecticides industry, fertilizer industry, pharmaceutical industry, general sewage treatment plants, special chemicals sector, paints and pigments sector, as well as other industries, are typically disposed of in the SIR landfills.^118^ Numerous studies have shown that LL is a significant source of developing contaminants, including PPCP waste.^119^ Leachate characteristics in the SIR landfill in Gujarat are shown in Table 3.^90,109,118^

**Table 3:** SIR leachate concentration

Parameter	Concentration (mg L^−1^)
pH	7.4–7.7
Color	—
SS	14 000–16 000
Chloride	13 000–63 000
Ammonium	16 000–22 000
TN	34 000–40 000
TP	30.1–35.8
COD	15 000–35 000
Oil	4.9–6.2
Cr_VI_^+^	0.628–0.902
Hg	—
Cd	0.318–0.573
BOD	11 000–30 000
Erythromycin	204 ng L^−1^
Glipizide	155 ng L^−1^
Carbamazepine (CBZ)	14 867 ng L^−1^

## 3 Recent advancements in landfill leachate treatment technologies and their advantages and disadvantages

Typically, leachates from MSW, both of which are ISW or SIR, are: (i) recyclable landfill, (ii) collected in on-site and off-site lagoons delivered directly to leachate treatment plant (LTP), (iii) treated biologically (aerobic or anaerobic) or (iv) exposed to conventional physical and chemical processes, *e.g.* chemical precipitation, coagulation/flocculation, sedimentation/air flotation and coating.

However, these methods have disadvantages, which are: (i) leachate recycling may affect landfill conditions, (ii) transfer of leachate is increasingly questioned and interferes with the proper functioning, and (iii) from a biological and physical and chemical point of view, conventional processes do not allow compliance with European standards of treated wastewater discharge standards.^53^ Hence, the global scientific community is focused on the development of effective LL treatment solutions.^96^

### 3.1 The technologies developed for leachate landfills

Treatment of leachate is often divided into three broad divisions: (1) the physical–chemical process, (2) the biological process, and (3) combination of the biological and physical–chemical processes.^120^ Coagulation, flocculation, precipitation chemistry, adsorption, filtration membranes, exchange ions, stripping air, advanced oxidation processes (AOPs), and electrochemistry are some of the physicochemical methods used to handle leachate. In order to remove non-biodegradable contaminants from LL, such as heavy metals, PCBs, and AOXs, physical–chemical processing procedures are often used.^121,122^

#### 3.1.1 Advanced oxidation processes (AOPs)

Due to the wide range of applications, the ability to compete using other pollutant degradation technologies, and high mineralization efficiency, AOP has recently become one of the most promising technologies for removing leachate contents. AOP converts emerging contaminants to less complex, non-toxic, and inorganic compounds ions, H_2_O, and CO_2_ to powerful oxidants known as free radicals or reactive oxygen materials. Ionizing radiation, ozonation, UV-based oxidation, Fenton and Fenton-like processes, electrochemical methods, ultrasound, photocatalysis, *etc.* combined techniques are some of the many AOPs strategies. Many detection studies have been conducted on PPCP from aqueous media by ozonation, Fenton, and UV-based oxidation procedures.^123^ Recently, much attention has been paid to oxidation—removal of PPCP from LLs. To remove PPCP, AOP was used alone or in combination with other chemical or biological methods. Fenton and ozone oxidation removal were investigated. Di(2-ethylhexyl) phthalate (DEHP) and nonylphenol (NP) from LL with autonomy variables such as reaction time 20–90 min, Fe(ii) dose of 0.5–2.55 g L^−1^, H_2_O_2_ dose of 5.0–25.5 g L^−1^, and pH 3–5 for Fenton oxidation and ozonation times of 10–130 min and pH 4–10 during ozone oxidation. NP was destroyed under most operating conditions due to high volatility; but short-circuit NP ethoxylates and NP carboxyethoxylates were the primary intermediates reported in the Fenton assay. During the Fenton oxidation and ozonation processes, DEHP removal efficiency was 90% and 50%. However, by-products of phthalic anhydride, benzoic acid, and pentanoic acid have been reported in ozonation processes.^124^ Concerns need to be raised about increased radiation doses by increasing the ultrasound power density and increasing the current density required to break down and mineralize PPCP extension. An increase in energy consumption and more expensive additional chemicals directly affect costs associated with AOP. Some of the main issues discussed in AOP are the efficacy of these methods for large-scale treatment of industrial wastewater and the financial potential of production costs related to such scaling-up operations. In rare cases, AOP may also include unidentified products, uncontrollable by-products that may act erratically, and pose a greater risk to human health than the parental bond.^125^

#### 3.1.2 Adsorption

The adsorption technique is a physicochemical treatment in which liquids are attracted to the surfaces of solid adsorbents to form bonds through physical or chemical bonds.^126,127^ Preparation of absorbent materials such as AC, biochar, and natural clay minerals requires temperature application. Proper preparation of adsorbents is important to improve the physicochemical properties to effectively adsorb pollutants through electrostatic attraction, complexation, hydrogen bonding, physical adsorption, and hydrophobic interactions.^128^ Several types of food waste in LL contents have been found to be effective adsorbents.^129^ The most commonly used adsorbent is activated carbon (AC), which has a relatively high surface area to adsorb pollutants such as heavy metals.^130,131^ AC is available in two forms: powder and granules. Granules-AC is used when the filtrate contains aromatic compounds and condensed structures. The effectivity in using powder-AC is its ability to remove 90% of COD, 40% of ammonium and 80–90% of heavy metals.^132^ One study reported high TSS, NH_3_–N, Zn, and Cu removals of 91%, 99%, 86%, and 100%, respectively, using coconut shell AC. The study showed that the rate of pollutant removal depends on the depth of the layer and the contact time of the adsorbent. Therefore, it is necessary to develop integrated and hybrid treatment processes for the efficient removal of heavy metals.^131^ For example, leachates that stabilized at 4500–8800 mg L^−1^ COD due to coagulation pre-treatment (50% COD removal) showed higher COD removal (80%) using AC adsorption.^133^ Similarly, other studies using banana leaves, sugarcane baguette,^130^ fish scales,^134^ and rice husks^135^ as adsorbents have been shown to be natural adsorbents effective in removing contaminants including COD, boron, NH_3_–N, phosphates and Fe. Although adsorption treatment has proven to be effective in recent years, high AC costs limit the use of this technology in developing countries.^136^ Many research studies recently developed mineralized adsorbents that have been shown to be effective in leachate treatment. However, AC sources need attention due to their worldwide availability as a natural mineral, their cost-effectiveness, and their ability to remove large amounts of pollutants in LL.^137^

#### 3.1.3 Filtration membrane

The membrane filtration technique is based on the principle of selective permeability of ions and molecules through thin film barriers. A selective barrier allows only certain molecules to pass through a composite fluid containing several species of contaminated ions and molecules that release pollutants in bulk liquids.^138^ Based on the particle size, the membrane filtration methods are divided into nanofiltration (NF), microfiltration (MF), ultrafiltration (UF), and reverse osmosis (RO).^139,140^ The UF technique is suitable for the separation of organic molecules present in the pre-treated filtrate, which has a relatively low COD of 1560 mg L^−1^ and BOD of 168 mg L^−1^ and the removal efficiencies were 46.7% and 22.1%.^141^ This was followed by an integrated study using the batch system NF and RO process pre-treatment of filtrate of coagulation/flocculation. The COD, TOC and NH_4_–N removal efficiencies were 28%, 59% and 8%, respectively. Subsequent application of NF and the RO process further improved the removal efficiency of the same pollutants by 95%, 93% and 89%, respectively.^142^ In the first stage of development, UF membrane filtration was used and has reached a very high removal of 98% TOC and some specific organic compounds, including acetone, methyl ketone, methyl chloride, phenol, toluene, *etc.*, up to 97%.^143^ The membrane filtration process is quite effective in removing contaminants from the pre-treated filtrate. However, the process faces several challenges in construction and operational requirements, such as the pre-treatment process, membrane fouling and fouling from organics, inorganics, purification of colloidal particles, molecules and heavy metal ions, very high energy consumption due to pressure pumping system, contaminants and the membrane after use, and improper disposal concentrated brine poses a threat to the environment and ecosystem as it contains toxic substances metals, halogenated by-products, antifouling, antifouling, *etc.*^144^

Treatment of leachate is biologically frequently used for the treatment of leachate with contaminants that have a high level of organic substances; treatment with technology has a lower effective cost, is simple to operate, and can be trusted as treatment of LL by the ratio of BOD/COD were high.^145^ Microorganisms, such as those involved in anaerobic digestion (AD), anaerobic filter (AF), up-flow anaerobic sludge ballet (UASB), and anaerobic ammonium oxidation (AAO), decompose organic molecules into biogas under anaerobic circumstances (anammox).^58^

Biological processing can be either aerobic or anaerobic, depending on the availability of oxygen.^146^ Aerated ponds, activated sludge aerobics, sequencing batch reactors (SBR), rotating biological contactors (RBC), moving bed biofilm reactors (MBBR), fluidized-bed biofilm reactors (FBBR), membrane biological reactors (MBR), fungal treatment, and constructed wetland (CW) are examples of environments where microorganisms degrade organic compounds into carbon dioxide and sludge under aerobic conditions.^48,147^ So many cutting-edge methods are needed to treat leachate while minimizing energy use, sludge creation, and toxin production. These methods also need to recover organic, inorganic, and xenobiotic substances in a safe phase and optimize their positive uses.^144^ The creation of an aerobic granular reactor (AGR) and constructed wetland microbial fuel cells (CW-MFC) for the treatment of LL is the result of recent improvements in biological processes over physical–chemical processes. These are potential methods for treating leachate because of their small footprint, lower energy need, power energy production, high microbial activity, long-term operational stability with greater elimination of organic pollutants, and tolerance to severe shock load.^148,149^

### 3.2 Constructed wetlands are advantageous over the others based on environmental attributes

Constructed wetlands (CWs) have been produced effectively in lab-scale experiments, pilot-scale projects, and full-scale field applications to treat landfill leachate (LL), demonstrating high efficiency in pollutant reduction.^150^ The concept of using wetlands for wastewater treatment dates back to the earliest periods of Chinese and Egyptian civilizations, where natural wetlands were employed to manage water pollution.^151^ CWs were first established for digesting various types of wastewater millennia ago, and in recent decades, they have evolved into fully designed systems.

CWs are engineered systems developed and operated to cleanse different forms of leachate, utilizing and optimizing natural environmental processes while meeting operational and maintenance needs for minimal upkeep systems.^152^ The type of constructed wetland produced is comparable to natural wetlands but involves the use of various media, including stone, sand, and soil, to create a matrix that supports plant growth and facilitates the treatment of leachate.^153^ A CW is a planned or regulated wastewater treatment system created utilizing natural processes that incorporate plants, media, and microbes.^154,155^ As the system's plants root, they transfer oxygen into the water, which serves as an energy source and catalyst for various microbial metabolic processes. Microorganisms degrade raw organic waste in the water into simpler substances that plants can then use as nutrients.^155^ This setup can be a cost-effective solution for wastewater treatment due to its minimal operational and maintenance expenses, as the processes occur naturally.

One of the most noticeable characteristics of CW and what sets them apart from uncultivated land is the presence of flora. The plants in the created wetland have a number of characteristics connected to the processing process, making them a crucial part of the CW's design.^156^ The most commonly used plants are *Typha latifolia*, *Phragmites australis*, *Juncus effusus*, *Iris pseudacorus*, *Glyceria maxima*, *Phragmites mauritianus*, *Cyperus papyrus*, *Typha angustifolia*, *Limnocharis flava*, *Eichhornia crassipes*, *Cyperus haspan*, *Acorus calamus*, *Carex rostrata*, *Sagittaria latifolia*, and *Thalia geniculate*.^157,158^ Although local plants have been used in CW for leachate, little research has been done on their utilization.^158–160^ Because native plants are more adapted to the area, using plants from beyond the CW site might hasten the harm to these plants.^158,161,162^ According to certain studies, the system-built CW for leachate can reduce NH_3_–N, NO_3_–N, NO_2_–N, and COD by 91.43, 94.19, 98.11, and 88.36% using *Canna indica*, *Phragmites australis*, and *Cyperus involucratus*.^163^ In another work, the author shows that the wetland plant *Scirpus validus* has the capacity to cure naproxen and CBZ in hydroponic circumstances. In testing conditions, 98% of naproxen and 74% of CBZ were removed successfully. According to the data, photodegradation and biodegradation contribute to naproxen removal, whereas plant uptake and assimilation contribute to CBZ removal.^115^

The treatment processes in CWs involve a combination of physical, chemical, and biological mechanisms.

#### 3.2.1 Physical mechanisms

Filtration and sedimentation: particulate matter is removed as water flows through the substrate. Larger particles settle due to gravity (sedimentation), while smaller particles are trapped by the substrate (filtration).^154^

#### 3.2.2 Chemical mechanisms

Adsorption: pollutants adhere to the surface of substrate particles through physical or chemical bonds; ion exchange: ions in the wastewater exchange with ions on the surface of the substrate particles; chemical precipitation: certain chemicals in the wastewater react to form insoluble compounds, which precipitate out of the water.^153^

#### 3.2.3 Biological mechanisms

Microbial degradation: microorganisms in the substrate and root zone degrade organic pollutants through aerobic and anaerobic processes; aerobic degradation: occurs in the presence of oxygen, often near the surface or root zone, where oxygen is supplied by plant roots or diffuses from the atmosphere; anaerobic degradation: occurs in the absence of oxygen, typically deeper in the substrate, where conditions are anoxic or anaerobic,^154^ nitrification and denitrification: nitrification is the aerobic conversion of ammonium to nitrate by nitrifying bacteria. Denitrification is the anaerobic conversion of nitrate to nitrogen gas by denitrifying bacteria, removing nitrogen from the wastewater;^152^ plant uptake: plants absorb nutrients such as nitrogen and phosphorus for growth, reducing the nutrient load in the water; and rhizofiltration: plants excrete root exudates that can bind and immobilize contaminants, enhancing microbial activity.^155^

**Fig. 1:**
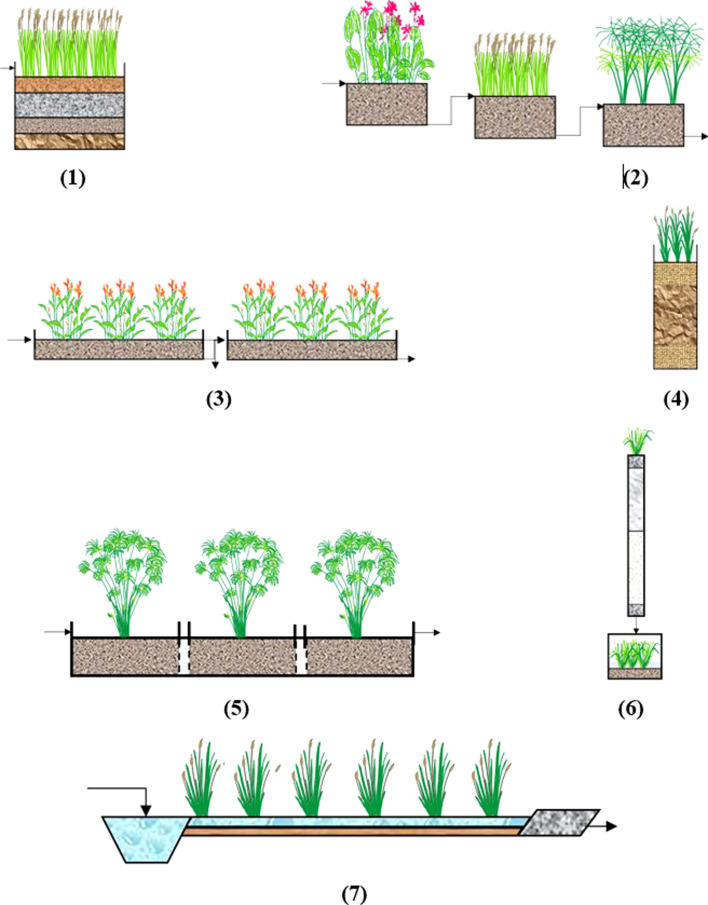
Design of CWs.

CWs leverage these mechanisms to treat wastewater efficiently while being cost-effective and environmentally sustainable. This approach minimizes operational and maintenance costs due to its reliance on natural processes, making CWs a viable solution for wastewater treatment (Fig. 1).^152^

CW method is mentioned by^164^ using *Typha angustifolia* plant for leachate treatment for 9 days can decrease COD, BOD, TSS, and TKN up to 75.81%, 69.84%, 91.16%, and 25.22%. CW modification using a combination of *Typha angustifolia* and *Cyperus papyrus* and bioaugmentation can improve CW performance in leachate treatment for COD, BOD, TSS, TN, Cd, and Hg, which were 80.47%, 84.05%, 80.05%, 75.58%, 99.96%, and 90%, respectively (Table 4).^170^

**Table 4:** Comparison of CW leachate treatment data

No.	Type of CW	Type of leachate	Operating condition	Influent parameter value (mg L^−1^)	Effluent parameter value (mg L^−1^)	References
1	Baffled-CW combined horizontal (H) and vertical (v) flow (F) *Phragmites australis*	SIR	HRT = 10 days (batch)	COD = 5163	COD = 1332.57	165
				TN = 439.2	TN = 119.81	
				TSS = 1994	TSS = 711.06	
				Cu^2+^ = 40.56	Cu^2+^ = 2.312	
				Ni^2+^ = 42.14	Ni^2+^ = 10.86	
2	Reedbeds-CW *Phragmites australis*	SIR	Intermittent influent flow	K = 174	K = 108	166
				Al = 5.7	Al = 1.1	
				Na = 1713	Na = 987	
				Cr = 0.01	Cr = <0.001	
				Ca = 138	Ca = 2.5	
				Li = 0.4	Li = 0.2	
3	HSF-CW	SIR	Continuous flow (1–55 L h^−1^)	Al = 17.27	Al = 0.33	167
	*Phragmites australis*			V = 0.14	V = 0.013	
4	VF-CW	MSW	Continuous flow (100 L per day)	AN = 320.35	AN = 9.15	168
	*Phragmites australis*			TN = 325.3	TN = 55.55	
				TP = 17.61	TP = 1.91	
5	TVF-CW	MSW	Continuous flow (10 L per day)	TN = 394.12	TN = 33.76	169
	*Canna indica*, *Phragmites australis*, & *Cyperus involucratus*			COD = 1022	COD = 119	
6	HSSF-CW	MSW	HRT = 6 days (batch)	COD = 9216	COD = 1799.89	170
	*Typha angustifolia* & *Cyperus papyrus*			BOD = 1140	BOD = 181.83	
				TSS = 120	TSS = 23.94	
				TN = 70	TN = 17.09	
7	HF-CW	MSW	HRT = 4 days (batch)	NH_4_ = 161	NH_4_ = 74%	171
	*Heliconia psittacorum*			COD = 691	COD = 84.7%	
8	VF-CW	MSW	Continuous flow (10 L per day)	COD = 378	COD = 226.8	172
	*Typha domingensis*			NH_4_N = 198	NH_4_N = 51.48	
9	HF-CW	MSW	Continuous flow (2.25 L per day)	COD = 660	COD = 396	173
	*Cyperus papyrus*			NH_4_N = 142	NH_4_N = 53.96	
10	Combination VF-SF	MSW	HRT = 23.3 days (batch)	COD = 838.5	COD = 258.5	174
				BOD = 274.6	BOD = 27.3	
				TN = 207.3	TN = 22	
				TP = 24.6	TP = 0	
				TSS = 432.6	TSS = 26	
11	HSF-CW	ISW	Continuous flow (60 L h^−1^)	Al = ±20	Al = ±0	175
	*Phragmites australis*, *Typha latifolia*, & *Sparganium erectum*			Cr = ±0.0015	Cr = n.d.	
				Ca = ±0.08	Ca = ±0.02	
				Mg = ±0.013	Mg = ±0.008	
				Na = ±0.35	Na = ±0.25	
				V = ±0.8	V = ±0.05	
				As = ±0.075	As = ±0.01	
12	SSF-CW	ISW	HRT = 7 days	COD = 3980	COD = 2626.8	176
	*Typha angustifolia*			BOD = 3465	BOD = 1282.1	
				VFAs = 707	VFAs = 438.34	
				TSS = 32.1	TSS = 22.47	
13	VF-CW	ISW	Continuous flow (0.008 m^3^ per m^2^ per day)	COD = 9740	COD = 1363.6	177
	*Phragmites australis*			DOC = 3535	DOC = 300	
				TSS = 1900	TSS = 1560	
				TN = 35.2	TN = 24.8	
				TP = 19	TP = 5	

The hydraulic loading rate is the volume of wastewater applied to the constructed wetland per unit area per unit time.^178–180^
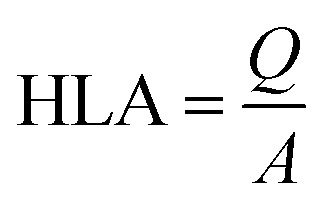
where, *Q* = flow rate of wastewater (m^3^ per day). *A* = area of the constructed wetland (m^2^).

The organic loading rate is the amount of organic matter applied to the constructed wetland per unit area per unit time.
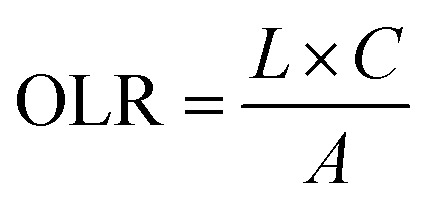
where, *L* = flow rate of wastewater (m^3^ per day). *C* = concentration of organic matter (*e.g.*, COD or BOD) in the wastewater (mg L^−1^). *A* = area of the constructed wetland (m^2^).

##### 3.2.3.1 Example calculations

(1) Hydraulic loading rate calculation

Example data:Flow rate (*Q*) = 100 m^3^ per dayArea of CW (*A*) = 500 m^2^

Calculation:
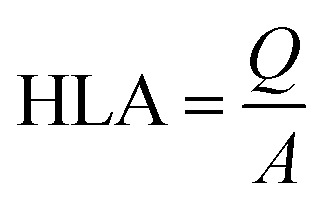

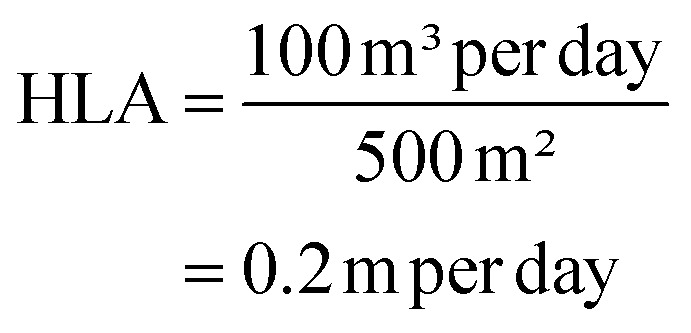


(2) Organic loading rate calculation

Example data:Flow rate (*L*) = 100 m^3^ per dayConcentration (*C*) = 300 mg L^−1^ (*e.g.*, COD)Area of CW (*A*) = 500 m^2^

Calculation:
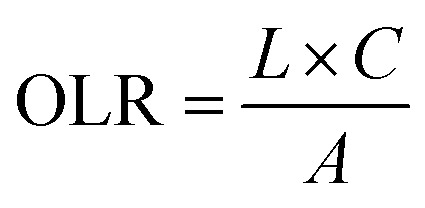

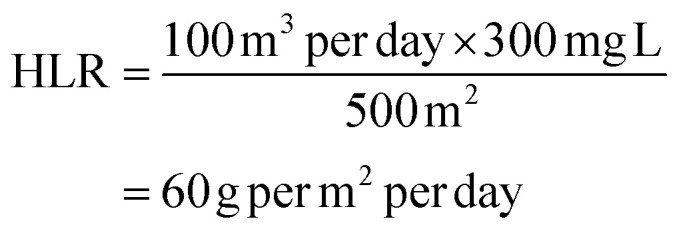


## 4 Integrated CW technologies for sustainable leachate treatment

**Fig. 2:**
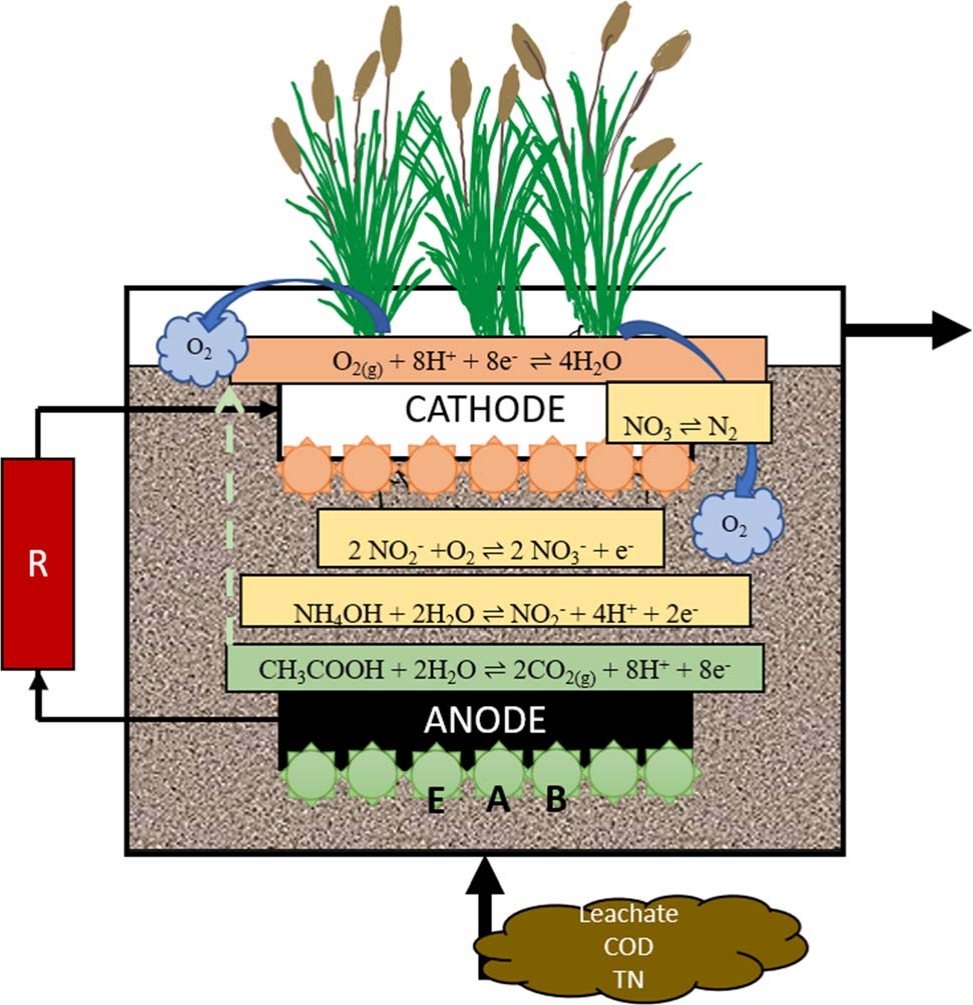
Mechanism of leachate treatment in CW-MFCs.

**Fig. 3:**
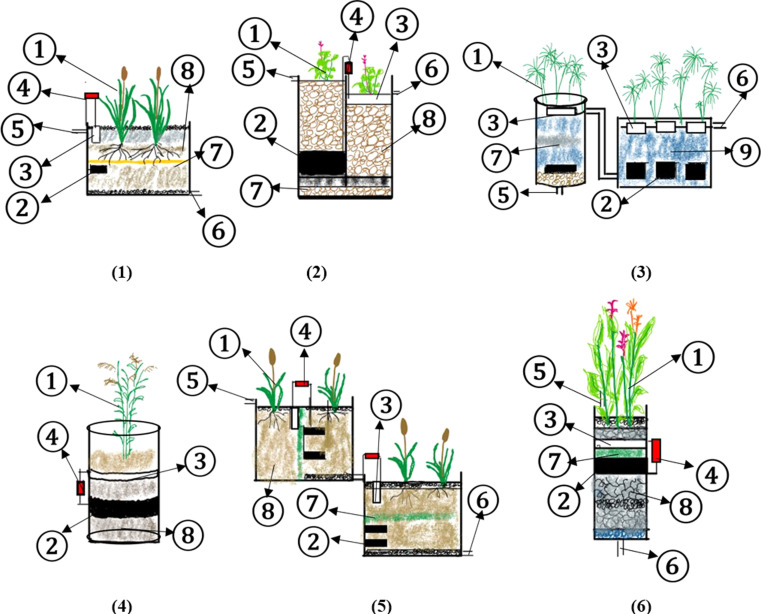
Reactor design from previous research ((1) plant, (2) anode, (3) cathode, (4) resistor, (5) influent, (6) effluent, (7) separator, (8) media, and (9) water).

The previous research studies had several differences, including the type of processing, type of electrode, type of plant, and HRT, which are shown in Table 5. The numbering in the table provides information for Fig. 2 and 3.

**Table 5:** Previous research on CW-MFCs

No.	Treatment type	Electrodes anode/cathode	Plant type	HRT	Voltage (V)	Power density	Current density	References
1	Synthetic WW	Graphite/magnesium	*Typha latifolia*	4 days	0.45–0.99 V	5.09 mW m^2^	7.11 mA m^2^	181
2	Synthetic WW	Graphite/magnesium	*Typha angustifolia*	4 days	0.79–1.34 V	181 mW m^2^	33.8 mW m^2^	10
3	Swine WW	Stainless-steel wire mesh wrapped with AC/carbon felt	*Canna indica*	2 days	598–713 mV	0.456 W m^3^	22.5 mA m^2^	182
4	Settle sewage	Granular AC/platinum coated with carbon	*Cyperus prolifer*	2 days	510 mV	229 mW m^3^	N/A	183
5	Swine WW	Stainless-steel mesh filled with charcoal/stainless-steel	*Cyperus* sp	2 days	0.58 V	56.9 mW m^3^	0.07 Am^3^	184
6	Urban WW	Graphite rods/PVC hole filled with graphite sticks	*Cyperus papyrus*	3 days	137.4 mV	0.93 mW m^2^	N/A	185
7	Synthetic eutrophication influent	Stainless-steel mesh/carbon felt	*Cyperus alternifolius*	2 days	125 mV	6.03 mW m^2^	N/A	186
8	Greywater	Graphite granules/graphite granules	*Phragmites australis*	2 days	150 mV	719.57 mW m^−3^	N/A	187
9	Boron in wastewater	Graphite nodes/graphite nodes	*Typha latifolia*	7 days	1600 mV	78 mW m^−2^	105 mA m^−2^	188
10	Leachate	Aluminium plate/aluminium plate	*Phragmites* sp	24 hours	45 mV	527 mW m^−2^	N/A	189
11	Leachate	Metallic aluminium/metallic aluminium	*Phragmites* sp	(N/A) minutes	Around 25 mV	3 mW m^−2^	Around 0.25 mA m^−3^	190
12	Leachate	Aluminium plate/aluminium plate	*Canna indica*	0.3 days	39–52 mV	20 mW m^−2^	0.4–0.5 mA m^−2^	191

### 4.1 CW-MFC synergistic effect and mechanism for leachate treatment

The most common substrates in leachate include ammonia, carbohydrate, and phosphate.^146,192–195^ The natural process that occurs in the wetland is self-purification.^196^ In the wetland, there are two kinds of agents that can have a big role: plants and bacteria in soil called rhizobacteria.^192,197^ The process includes sedimentation, filtration, reduction–oxidation (redox), and plant uptake.^198^ The rhizobacteria play an essential role in the elimination of pollutants through the redox process.^199^ Natural purification in the environment can treat water quality with slow processes; therefore, modifications have been performed by many researchers.^200^ In CW, the rhizobacteria will utilize some acceptors and electron donors from pollutants in the water for their growth.^201^ The electron acceptors in CW, as time goes by, will be limited due to the anaerobic conditions. The removal performance will reduce because of the unavailable suitable oxygen or electron acceptors. In MFC, an anode is used as an electron acceptor and a cathode for an electron donor.^202^ The mechanism of leachate treatment in CW-MFCs is shown in Fig. 3.

The mechanism for leachate treatment for organic compounds, nitrogen removal, and heavy metals occurs through several processes, which are physical, chemical, biological, and electrochemical and microorganism synergism processes.^203^ Pollutants in leachate will be decomposed and oxidized by the anode under anoxic or anaerobic conditions. In the anode, electrons will be generated and moved by an external wire to the cathode. The oxidation–reduction process during leachate treatment is shown in eqn (1) and (2).^204^

Oxidation that occurs in the anode

1 HOOCOOH_(aq)_ + H_2_O ⇌ 2CO_2(g)_ + 2H^+^ + 2e^−^

Reduction that occurs in the cathode

2 O_2(g)_ + 4H^+^ + 4e^−^ ⇌ 2H_2_O

The EAB accepts the electron from the electrode by direct electron transfer *via* the oxidation of hydrogen and other compounds.^205^ Then, protons are transferred through diffusion towards the cathode, and the protons and electrons eventually reduce with oxygen secreted from the root plant or NO_3_–N on electron acceptors, which is the cathode. The chemical energy will be converted into electrical energy in the cathode.^206^

High voltage and power density in CW-MFC can be reached through several factors; the maximum treatment and electrical efficiency in CW-MFC depends on the organic loading rate (OLR). The average and maximum voltages recorded from previous research reported that the anode region removed organics satisfactorily while the cathode region remained aerobic to carry out oxygen reduction reactions at low OL where a closed-loop HSSF CW-MFC showed satisfactory performance (98–99%) at low volume OLR (0.15 and 0.30 kg COD per m^3^ per day). However, at high volume OLR (0.52 kg COD per m^3^ per day), the performance decreased to 95.4%. Additionally, a significant performance improvement of 37.7% was observed in the closed-loop design compared to the open-loop HSSF CW MFC at a high volume OLR of 0.52 kg COD per m^3^ per day.^207^

The other factor that increases voltage and power density. Plants play an important role in the CW-MFC system because they release oxygen and release exudates into the rhizosphere through their roots, supporting the biogeochemical cycling of various elements. They provide a large surface area for microbial colonization and influence the microbiota associated with the rhizosphere. The dense and fine root network has a filtration effect that improves the quality of treated water. The difference between each plant from the previous study shows the different electricity production and the best plants need to be considered. The role of evapotranspiration in stress generation was monitored by introducing CW-MFC planted with *Phragmites australis* at a pilot scale. Larger voltage fluctuations were found to occur during warm days than during cold seasons due to higher evapotranspiration.^208^

The type of electrode material can influence the high-power density and voltage; microbial electron transfer depends on the biocompatibility of the materials. The structural and functional properties of each material determine microbial compatibility, surface area and porosity, as well as habitat quality.^181^ Carbon-based materials have excellent biocompatibility and electrical conductivity. Therefore, carbon-based materials such as graphite, carbon cloth or felt, and activated carbon are commonly used as electrodes in CW-MFCs. In addition, the electrode positioning affects the current output of the CW-MFC. For its efficient functioning, anaerobic and aerobic conditions are required in the anodic and cathodic regimes, respectively. Additionally, it is important to maintain a minimum distance between the electrodes to reduce internal resistance.^209^ reported that the optimal position is to place the cathode 1–2 cm above the water surface.

The quantity of the electrode can increase the electricity and power density production. The use of multiple electrodes in a CW-MFC system is directly related to the increase in the total surface area of the electrodes relative to the total volume of the anode or cathode. Such an arrangement ensures maximum electron transfer to and from the electrode. Placing multiple cathodes at appropriate locations changes the redox potential within the treatment bed and reduces energy loss. The maximum power densities of systems with parallel electrodes and ventilation, recirculation, and both ventilation and recirculation were 1.55 mW m^−2^, 3.09 mW m^−2^, and 7.99 mW m^−2^, respectively. In a previous study, the output of several anodes embedded in CW was investigated by MFC in series and parallel circuits.^210^

With the increased surface area, decreased charge-transfer resistance, and increased bacterial loading mentioned above. Previous research reported on an H-shaped two-chamber MFC equipped with a hybrid anode made of graphene (G) and a conducting polymer, poly(3,4-ethylenedioxythiophene) (PEDOT), (G/PEDOT) using *in situ* electro polymerization. Comparing the G/PEDOT hybrid anode to the CP, CP/G, or CP/PEDOT anodes, the former generated more power and had a larger loading of bacteria. The MFCs using the G/PEDOT hybrid anode ought to have a greater output power density than those made using the other three anodes. The results of this work suggest that the G/PEDOT hybrid may make a good anode material for MFCs.^211^ According to similar findings, GF anode modified with PEDOT shows promise as an anode material for MFC technology, both in terms of performance and scaling up.^212^ Furthermore, the structure and design of the electrodes have a significant impact on the anode's capabilities. Over planar electrodes made of fabric and plate construction, felt anodes were apparent as potential anodes. The felt structure's shape clearly acknowledged the ability to build biofilms and transport of electrons. Overall, the felt structural structure and PEDOT combined to secure the GF-P as a viable anode for CW-MFC applications.^213^

Biological processes during leachate treatment in CW-MFCs are biochemical from HRT, plant uptake (the biodegradation of fillers, microorganisms and rhizosphere), and also the conversion process by microorganisms for pollutants on the electrode surface, which was affected by electrode material types.^41,204,214^ Plants play an essential role in CW-MFC systems because they liberate oxygen and exudates through the root system into the rhizosphere zone and assist in the occurrence of biogeochemical cycling of various elements.^196^ The plants have been shown to improve bacterial activity to decompose organic compounds.^24^ The plant has a large specific surface area that can enhance the adsorption of electron-transfer mediators, providing advantages to the CW-MFC systems.^11,27^ Both electricity generation and water purification rely on the microbial oxidation of inorganic and organic matter in leachate.^184^ The CW-MFC can significantly improve the efficiency compared to the MFC or a CW.^181,207,215,216^

### 4.2 Effect of different design parameters

The topic of CW-MFC has recently been updated. During the past ten years, a lot of research has been done on constructed wetlands (CWs) integrated with bio electrochemical systems (BESs). These systems have been dubbed electro-wetlands, electroactive wetlands, microbial electrochemical technologies-based constructed wetlands, and constructed wetland-microbial fuel cells (CW-MFC).^217^

From the data collected in the table, the reactor design for each research study is shown in Fig. 3.

**Fig. 4:**
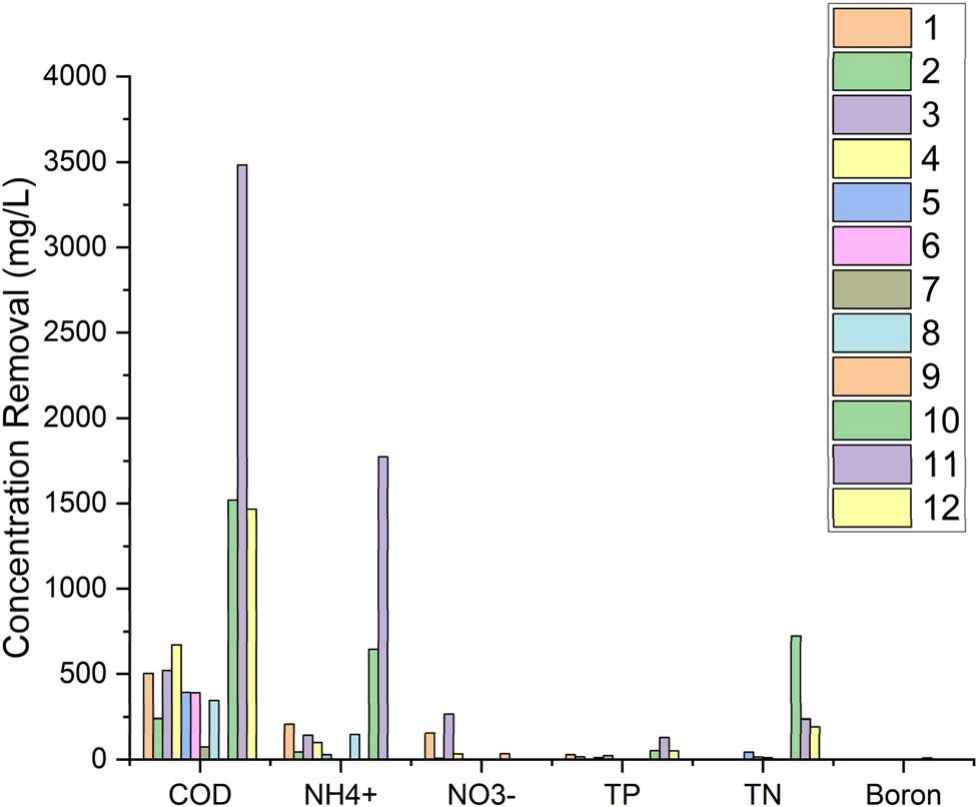
Removal concentration based on previous research.

From the results of data collection obtained in the table, the concentration of removal for each research is shown in Fig. 4.

In the numbering above, No. 1 refers to that in Table 1. Number 1, which was carried out by Yakar *et al.*,^181^ (2018), shows synthetic WW processing with anode graphite and cathode using magnesium, plants *Typha latifolia* for 4 days can produce a voltage of around 0.45–0.99 V, power density 5.09 mW m^−2^, current density 7.11 mA m^−2^, with removal efficiencies, COD 90.6%, NH_4_^+^ 92.6%, NO_3_^−^ 81.47%, and TP 96.7%. Other studies have shown different results due to different plant species, HRT, electrode type, CW-MFCs design, and other variables.

The material or conductivity and surface energy of the electrode or conductivity is one of the important factors for the treatment process, stability, price, and bioelectricity generation in CW-MFC.^181,196,218–220^ The porosity and surface area, habitat quality, microbial affinity and biocompatibility of the material needed for microbial electron transfer are important.^181,221^ The previous studies have been conducted using granular activated carbon (GAC),^183^ graphite rod (GR),^222^ graphite gravel (GG),^204,223^ graphite plates (GP),^224,225^ graphite felt (GF),^226^ carbon fiber felt (CFF),^227^ carbon fiber brushes (CFB),^224^ carbon felt (CF),^226^ activated carbon (AC),^228^ carbon nanotube (CNT),^229^ carbon cloth (CC),^183^ metal nanoparticles,^230,231^ and graphene oxide,^232^ as electrode materials.

Metals have a large specific surface area that can increase electrical conductivity and harvest maximum electrons;^220^ in addition, carbon also has good electrical conductivity and biocompatibility.^196^ Graphite and carbon are low-cost materials and have strong chemical stability.^233^ Some researchers have developed and modified the electrode materials.^220^ The combination of metals modified with carbon materials^234^ in cathodes has the function of reducing electron acceptors, while anion materials should be resistant to abiotic or biotic degradation because the bacteria should grow on the surface and allow high electron transfer rates.^205^

Factors that affect pollutant degradation are based on the electrode size and type of electrode in CW-MFCs.^220^ Nanoporous and microporous materials will make a high surface area suitable for the growth of Electroactive Bacteria (EAB).^205^ Modification to activate the high surface area of the electrode can be used in pre-treatment.^235^ The pre-treatment for enhancing the surface area of the electrode is heat,^236^ adding redox mediators,^237^ modifications with other materials, such as zeolite clay composites with metals^238^ or graphene oxide (GO),^232^ and doping superficial nitrogen groups on the anode surface.^235^

### 4.3 Leachate treatment

#### 4.3.1 Nitrogen

The process of nitrogen removal occurs through two major stages, known as nitrification and denitrification. In the nitrification process, aerobic bacteria are used and an electron acceptor is required to convert ammonia into nitrite and nitrate. Then, in the denitrification process, anaerobic bacteria are used and an electron donor is required for the conversion of nitrate into nitrogen gas (N_2_).^239^ The rate of the nitrogen process depends on the availability of electron donors that are COD and NH_4_^+^ and electron acceptors O_2_ and NO_3_–N in CW-MFCs. The majority of researchers reported an improvement in CW-MFCs compared to CWs for the removal of nitrogen, about >10%,^28,182,240,241^ although a minority of researchers did report a reduction.^187,206^ In MFCs, the cathode is a nitrifying bacteria zone, and anode zone can increase the community of anaerobic ammonium oxidation (anammox) and denitrifying.^206,241–244^ The anammox process also occurs, in which ammonia is autotrophically oxidised into N_2_ when NO_3_–N under anaerobic conditions is used as the electron acceptor.^245^ In addition, microbial ammonia oxidation, nitrification, denitrification, adsorption, and plant uptake N occur in CW.^192,246^ Overall, it can be concluded that CW-MFCs are efficient in terms of nutrient removal; however, the pollutant removal performance highly depends on the design of the system.

#### 4.3.2 Phosphate

Polluted water usually contains 5–20 grams of phosphate and other substances.^247^ In the natural environment and leachate, P exists in various forms, such as *ortho*-phosphate (containing one phosphate unit), poly-phosphate, pyro-phosphate, *meta*-phosphate and their organic complexes.^248^ Phosphate can be removed by physicochemical and biological processes. Physicochemical (adsorption and electrochemical) processes in CW-MFCs are the most influential processes for phosphate.^24,249,250^ The media in CW-MFC, such as soil, gravel, and other sediments, will absorb and adsorb phosphorus.^251^ In previous research conducted using CW-MFC, the phosphorus parameter was reduced to 100%.^252^ Besides physicochemical processes, the plant plays an essential role in uptaking phosphorus for their growth. The macrophyte uptake of the phosphorus was usually highest during the growing season.^251,253^ Moreover, phosphate uptake is affected by pH; absorption decreases in acidic and relatively alkaline environments.^254^

#### 4.3.3 Chemical oxygen demand (COD)

Oxygen is used to degrade organic material for electrochemically active bacteria (EAB) activity and growth in CW-MFCs. In MFCs, organic carbon will go to the anode zone and the rest of it will move to the cathode zone with less oxygen.^255–257^ The COD removal can achieve greater than 95% with 44.6 mW m^−2^. Other factors that affect the removal of COD are C/N ratio, pH, initial nutrient concentration, chemical species, Hydraulic Retention Time (HRT) and salinity.^183,241,258–267^ Long HRT will make CW-MFCs to anaerobic conditions that effect on DO concentration and activities of microbial.^241,262^ Research have conducted that maximum level of HRT is 3 days and will decline more than the days.^268^

#### 4.3.4 Heavy metals

Heavy metals in waste can also be removed with MFC through a bioelectrochemical process.^269^ Heavy metal removal occurs from H_2_O_2_ electrogeneration at the cathode, which is driven by heavy metal-reducing bacteria.^269^ Thus, the integrated CW-MFC can increase the removal of heavy metals apart from plant extraction as well as from bioelectrochemical processes. Removal of Zn and Ni in leachate using CW-MFC reached up to 80%, with *Iris pseudacorus* and water hyacinth plants yielding up to 534.30 mV.^270^ Some heavy metals in leachate-containing groups have high redox potentials that could be utilized as electron acceptors in order to get precipitated and reduced.^271^ In general, the anode portion is used to degrade pollutants/organic through biocatalytic oxidation, whereas the cathode is mainly an enclosure for a CW-MFC circuit or a destination for electrons and protons. Compounds with high redox potentials, such as some heavy metals, can serve as cathodic electron acceptors in CW-MFCs.^272^ High removal of Zn, Pb, and Cr by CW-MFC has been reported, with heavy metal concentrations 5–80 mg L^−1^ reaching removal efficiency up to 99%.^273–276^

## 5 Future perspective

There are many ways to use it to develop and comprehend CW technology, such as identifying root secretions, where they can in fact, serve as electron donors for plant species, enriching the rhizosphere to increase the efficiency of leachate processing, and numerous studies developing it to produce additional benefits, such as bioelectricity.^277^ In CW-MFCs, the anode and cathode potential still have to be thoroughly investigated for their potential influence. In addition, the fundamental difficulty with CW-MFC is that up until now, all of the research has been done on a laboratory scale. The energy generation, operating parameters, design configuration, electrode material and size, and other scaling-up issues are important, which is why CW-MFC is still being developed on a laboratory scale.^217^ In the development of MFC-CW, the entire Spanish application was upgraded, even if it is still in its infancy sewage clean-up. There are several issues with the system's design,^208,278–280^ operation and power output,^281,282^ electrode materials,^283,284^ the function of plants,^208,285^ enhanced biodegradation,^286,287^ new contaminants,^288,289^ and biosensing development,^290,291^ among others. Some review publications, such as those in ref. 292–295, presented an updated evaluation and in-depth study of CW-MFC in a timely way. Theoretically, other avenues of inquiry may focus on both aspects and practical investigations. To be more effective than other leachate treatment techniques, CW-MFC must be scaled up and deployed in practice and on a large scale. Several studies on CW-MFC have yielded a lot of information on the advantages and disadvantages of CW-MFC as a treatment technology, and these have been summarized in Table 6.

**Table 6:** The advantages and disadvantages of CW-MFCs

No.	Research application	Advantages found from the research	Disadvantages	Source
1	CW-MFC for WW treatment	Using activated carbon as the filler can reduce the clogging	Clogging without filler in CW-MFC	296
2	Research about microbial population during MFC start-up	The addition of acetate conditions the active bacteria so they are expected to become stable	Voltage instability	297
3	Maximizing energy harvesting by adjusting duty cycle value	Systems using capacitors can sustain energy losses	High internal resistance can cause high energy loss	298
4	CW-MFC to enhance NH_4_^+^ removal	Microbial activity and NH_4_^+^ removal can be maximized by increasing the voltage	Voltage must be applied to increase electron transfer and microbial activity	299
5	Effects of different connection modes and cathode conditions in CW-MFC	COD removal can be eliminated up to around 70% even with low power density	The highest power density of CW-MFC depends on both the coulombic efficiency and net energy recovery	300
6	Effect of electrode material and substrate	Increasing the substrate concentration enhanced the power density	Substrate limitation	301
7	Relationship electricity performance CW-MFC in non-growing seasons	In the growing season and suitable weather, the CW-MFC configuration will best	In low temperatures, the plant and microbial activity will impended	302

The disadvantage of CW-MFC from previous studies can be considered or improved by future studies using CW-MFC for LL treatment. From the limitations and advantages of CW-MFC, in the future, these limitations can be researched or re-examined for handling, and these advantages really make a big contribution to future researchers. Several recommendations have been made to promote the adoption and widespread use of CW-MFC for wastewater treatment and energy generation. Investigating alternative electrode materials and cost-effective fabrication techniques, investigating diverse applications beyond energy generation, conducting comprehensive economic viability and sustainability assessments, improving stability and longevity through electrode modifications, and addressing scalability challenges are among the recommendations. It is also proposed that CW-MFC technologies be integrated with membrane filtering process techniques. Implementing these ideas has the potential to make considerable progress in terms of increasing efficiency, lowering costs, and improving environmental sustainability. Thorough economic viability analyses to compare the costs of CW-MFC technology to those of conventional wastewater treatment methods have to be conducted. Life Cycle evaluation (LCA) and exergy analysis are two sustainability evaluation methodologies that may examine the environmental, economic, and social aspects of CW-MFC systems. LCA studies must be refined and expanded in the future to analyse new CW-MFC designs, materials, and operating conditions for future energy storage applications. Furthermore, the use of energy analysis to evaluate energy conversion efficiency and identify potential for improvement is advised. CW-MFCs can unlock their full potential by bridging the 4Es (Energy, Environment, Efficiency, and Economics) of bioenergy systems and comprehensively assessing their environmental effects, providing more sustainable and efficient solutions for wastewater treatment and other environmental applications.

## 6 Conclusions

As a result, future researchers and environmental engineers may find it challenging to achieve significant economic sustainability by optimising those factors (type of electrode material, plants, and OLR). An exhaustive effort has been made in the current study to make the CBA model as practical as possible by using raw domestic wastewater instead of synthetic wastewater and carefully analysing local factors, such as labour cost, cost of land, electricity tariff, cost of effluent quality check, and so on. However, as scaling up bioelectrochemical systems imposes a nonlinear response comparable to lab-scale reactors, the investment cost, operation and maintenance cost, and cost of power output may fluctuate throughout the installation and operation of field-scale reactors. The correlation matrix revealed a positive relationship between electricity and pollutant treatment, implying that a reactor with a greater power output will create higher-quality effluent. Future research may focus on additional advantageous uses of treated wastewater following rigorous evaluation of effluent quality criteria in order to earn more money from industrial and commercial reuse of treated wastewater. CW-MFC can be used as a sustainable treatment process for leachate along with electricity generation and environmentally friendly removal of several harmful compounds in wastewater. The power generation in CW-MFC ranges from 25–1600 mV. The removal efficiency of leachate treatment using CW-MFC from the previous study report was 90.6% for COD, 92.6% for ammonia, and 96.7% for TP. So far, mixed aspects of the CW-MFC-related design and process have been investigated on a laboratory scale. For field applications, CW-MFC needs to be further innovatively developed and optimization of technology is required by considering several major limiting parameters such as the PPCPs, plasticizer or BPA, and heavy metals.

## Data availability

The necessary data used in the manuscript are already present in the manuscript.

## Author contributions

Isni Arliyani: conceptualization, data curation, formal analysis, funding acquisition, investigation, methodology, project administration, resources, software, supervision, validation, visualization, writing – original draft, writing – review & editing. MD Tabish Noori: conceptualization, data curation, formal analysis, funding acquisition, investigation, methodology, project administration, resources, software, supervision, validation, visualization, writing – original draft, writing – review & editing. Muhammad Imam Ammarullah: conceptualization, data curation, formal analysis, funding acquisition, investigation, methodology, project administration, resources, software, supervision, validation, visualization, writing – original draft, writing – review & editing. Bieby Voijant Tangahu: conceptualization, data curation, formal analysis, funding acquisition, investigation, methodology, project administration, resources, software, supervision, validation, visualization, writing – original draft, writing – review & editing. Sarwoko Mangkoedihardjo: conceptualization, data curation, formal analysis, funding acquisition, investigation, methodology, project administration, resources, software, supervision, validation, visualization, writing – original draft, writing – review & editing. Booki Min: conceptualization, data curation, formal analysis, funding acquisition, investigation, methodology, project administration, resources, software, supervision, validation, visualization, writing – original draft, writing – review & editing.

## Conflicts of interest

The authors declare no conflict of interest.
